# Development and validation of the NCC‐BC‐A scale to assess patient‐reported outcomes for breast cancer patients in China

**DOI:** 10.1002/cai2.141

**Published:** 2024-10-18

**Authors:** Fei Ma, Xiaoyan Yan, Xiuwen Guan, Tianmou Liu, PRO‐BC China Standards Committee

**Affiliations:** ^1^ National Cancer Center/National Clinical Research Center for Cancer/Cancer Hospital Chinese Academy of Medical Sciences and Peking Union Medical College Beijing China; ^2^ Clinical Research Institute, Institute of Advanced Clinical Medicine Peking University Beijing China; ^3^ Department of Epidemiology and Biostatistics, School of Public Health Peking University Beijing China

**Keywords:** breast cancer, patient‐reported outcome, quality of life

## Abstract

**Background:**

The commonly used international patient‐reported outcome scales for breast cancer were developed before the advent of multiple targeted therapies and immunotherapies, rendering them potentially insufficient for current clinical practices. Therefore, it is necessary to develop a specific patient‐reported outcome scale tailored for breast cancer patients in China to optimize the management model for these patients.

**Methods:**

A comprehensive literature search was performed in the PubMed, Embase, Wanfang, and CNKI databases to extract dimensions and items for a potential patient‐reported outcome scale. The Delphi method was used to modify, add, subtract, and adjust the language of items until the experts reached a consensus on the first draft. This draft was further refined using a cognitive test and a presurvey. The optimized scale was used for a formal survey, and the items were further analyzed and screened using metrics such as the coefficient of variation, correlation coefficient, internal item consistency, factor analysis, reliability, and validity.

**Results:**

A total of 10,954 articles were analyzed, and 237 were used to create a pool of 277 patient‐reported outcome items. Through two rounds of Delphi expert consultation, the experts' authority coefficients were 0.739 and 0.826. After a cognitive test, several items were adjusted to enhance understanding. Further adjustments were made following a presurvey of 200 advanced breast cancer patients, resulting in a 38‐item patient‐reported outcomes scale, termed NCC‐BC‐A. In the national formal survey, 588 advanced breast cancer patients participated. Principal component analysis showed good consistency among the items and sufficient difference between the dimensions. The results were normally distributed with good variation. The Cronbach's *α* coefficient of the scale was 0.925 and the test–retest reliability was 0.9041.

**Conclusion:**

The NCC‐BC‐A scale has high validity and reliability. It comprehensively considered the characteristics of systemic treatment for breast cancer, and the specific context within China. Its implementation may help clinicians to pay more attention to quality of life of breast cancer patients and to optimize the system for managing this condition.

## Graphical abstract

This study developed a NCC‐BC‐A scale with relatively high validity and reliability for breast cancer (BC) patients. NCC‐BC‐A scale fully considered the characteristics of systemic treatment status for BC and the realistic situation in China, which may help clinicians to pay more attention to QOL of BC patients and to optimize management system for BC.

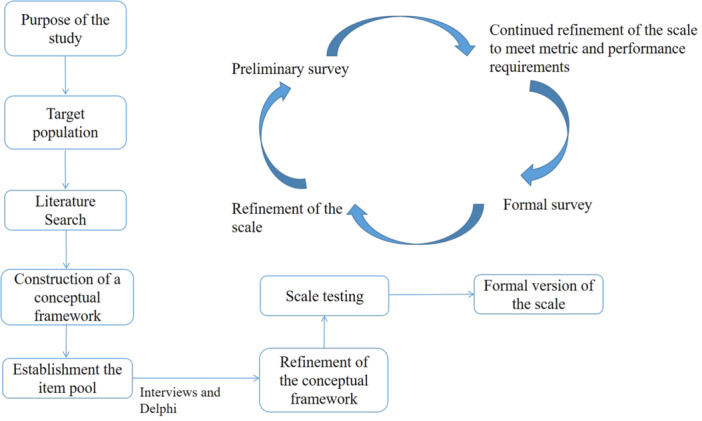

**Abbreviations**
BC: breast cancer
CDE: Center for Drug Evaluation
EORTC: European Organization for Research and Treatment of Cancer
FACT‐B: Functional Assessment of Cancer Therapy‐Breast
PRO: patient‐reported outcome
QLQ‐BR23: Quality of Life Questionnaire Core Breast Cancer‐specific supplement
QLQ‐C30: Quality of Life Questionnaire Core 30
QOL: quality of life

## 1 INTRODUCTION

Breast cancer (BC) is the most commonly diagnosed malignancy worldwide, with an estimated 2,261,419 new cases in 2020 (11.7% of all cancers) and 684,996 related deaths (6.9% of all cancer‐related deaths) [1]. Chinese women constitute approximately 18% of all new BC cases worldwide [2]. The actual cancer burden in China is probably underestimated due to limited registry coverage, which covered only about 32% of the population in 2019 [3]. BC treatment involves multiple disciplines, including local treatment (surgery and radiotherapy) and systemic treatment (endocrine therapy, chemotherapy, targeted therapy, and immunotherapy) [4, 5, 6, 7, 8]. While 70%–80% of patients with early‐stage nonmetastatic BC can be cured, no curative treatment currently exist for advanced BC with distant organ metastasis [4]. Patients with metastatic BC therefore receive systemic treatment to relieve symptoms and increase their quality‐adjusted life years [4]. The approval of new agents and the application of new treatment strategies have significantly improved the survival rates of advanced BC patients in recent decades. Therefore, improving the quality of life (QOL) of these patients has become an important goal in both clinical practice and clinical research [9].

A patient‐reported outcome (PRO) is defined as any outcome derived from patients' assessments of their feelings about their disease, QOL, adverse effects, or functional status that is directly reported by patients and not modified or interpreted by others. It is considered the “gold standard” for direct data collections in areas such as health‐related QOL, treatment preferences, and satisfaction with care [10, 11, 12]. Clinicians may fail to detect severe symptoms in a timely manner and may underestimate some symptoms during routine consultations. However, the application of PROs can improve symptom awareness and management, improve communication with physicians, decrease unplanned healthcare utilization, reduce emergency room visits, enhance QOL, and prolong survival time.

The commonly used international PRO scales for BC include the European Organization for Research and Treatment of Cancer (EORTC) Quality of Life Questionnaire Core 30 (QLQ‐C30), the EORTC Quality of Life Questionnaire Core Breast Cancer‐specific supplement (QLQ‐BR23) [13, 14, 15], and the Functional Assessment of Cancer Therapy‐Breast (FACT‐B) [16]. The EORTC QLQ‐C30 is suitable for the assessment of PROs for various cancers and is not specific to BC. In contrast, the EORTC QLQ‐BR23 and FACT‐B are BC‐specific scales designed to evaluate PROs among BC patients undergoing both local and systemic treatments [16]. However, the QOL in BC patients varies greatly with the advent of new treatment strategies, such as immune checkpoint inhibitors, CDK4/6 inhibitors, and antibody‐drug conjugates. Additionally, these scales were developed before these newer treatment strategies became common, which may make them less suitable in the current context. There are also substantial linguistic and cultural differences between China and Western countries, where these questionnaires were originally developed and validated. Consequently, it is likely that the existing PRO scales for BC do not completely capture the QOL of Chinese patients with BC.

This multicenter, cross‐sectional study aimed to develop and validate the reliability and validity of a specific PRO scale (NCC‐BC‐A scale) for BC patients in China. This PRO scale is intended to comprehensively assess the QOL and symptomatic adverse effects for BC patients in China, potentially helping clinicians in optimizing the management system for BC.

## 2 METHODS

### 2.1 Study design

**Figure 1:**
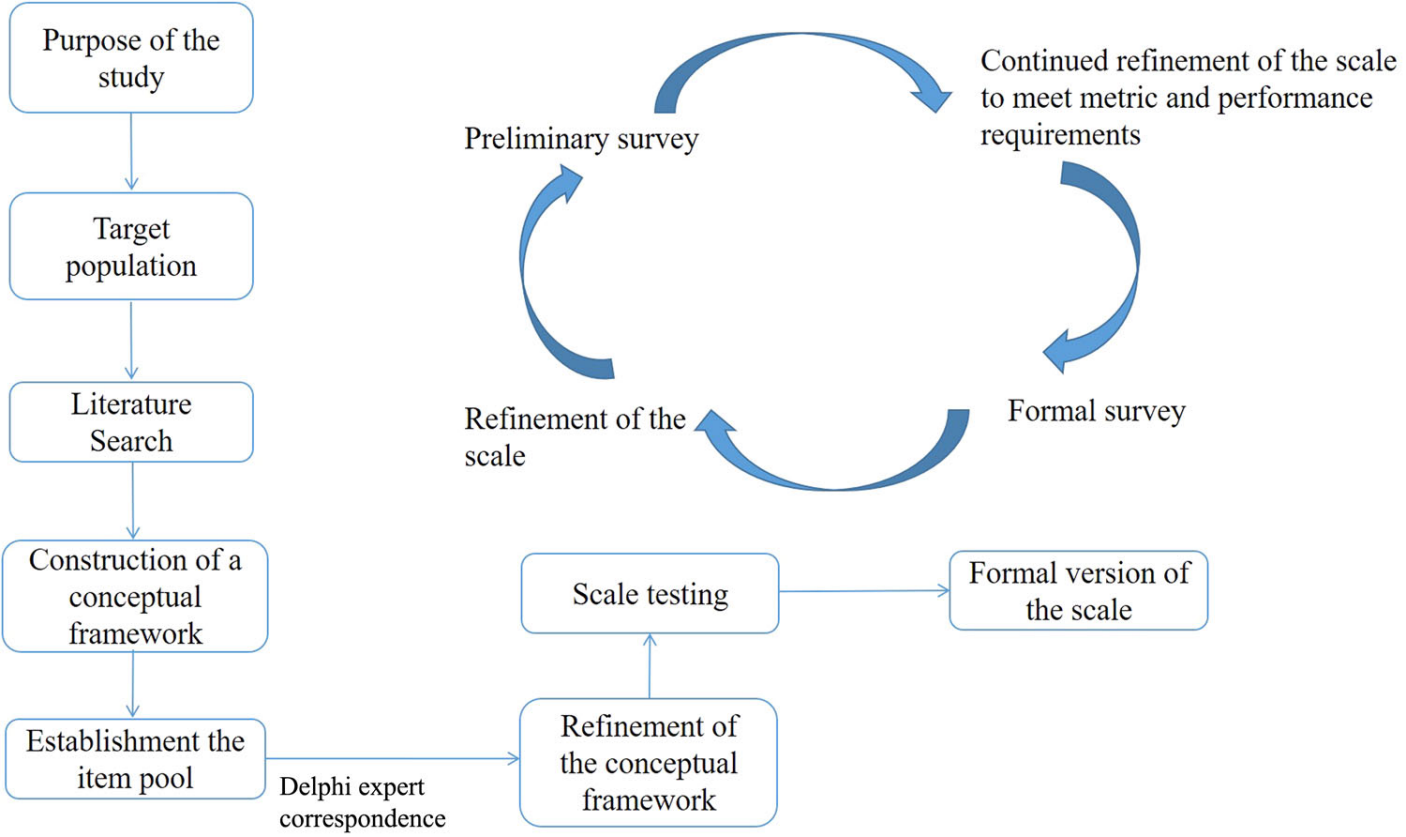
Roadmap showing the process of creating the NCC‐BC‐A Scale.

Our new PRO scale, the NCC‐BC‐A scale, is based on a theoretical model with the three health‐related domains outlined by the Medical Outcomes Study (MOS) framework: physiological, psychological, and social domains [17]. We adhered to the PRO development process advocated by the Center for Drug Evaluation (CDE) of the National Medical Products Administration in China [18] (Figure 1). This process involves: (1) constructing a conceptual framework, (2) establishing an item pool, (3) item scaling, (4) conducting expert interviews or expert surveys, (5) conducting preliminary and formal surveys, (6) validating the conceptual framework (reliability and validity), and (7) drafting the scale instructions.

This study involved three phases: (1) item generation, (2) item reduction, and (3) validation of the reliability and validity of the scale. A QOL standardized assessment project collaborative group consisting of oncologists specializing in BC, psychologists, nurses, statisticians, and representative BC patients, supervised and reviewed this study.

The study was approved by the Ethics Committee of National Cancer Center/Cancer Hospital, Chinese Academy of Medical Sciences and Peking Union Medical College (approval number 22/245‐3447). All participating doctors, nurses, and patients provided written informed consent.

### 2.2 The first phase: Item generation

**Figure 2:**
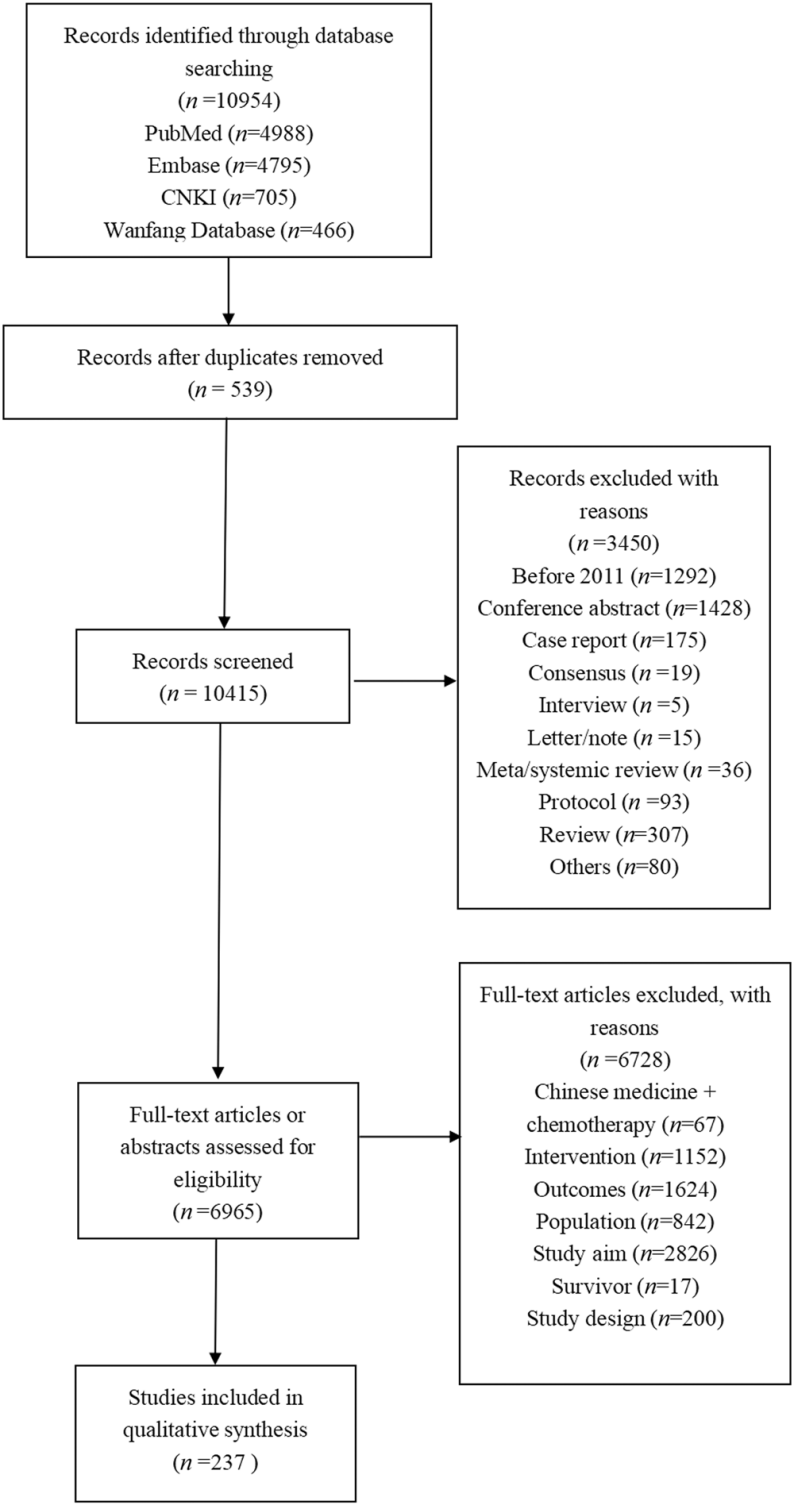
Literature screening criteria and process.

The purpose of this phase was to create a preliminary list of items. First, we developed a search strategy using search terms such as “breast cancer,” “metastatic,” “recurrence,” “advanced,” “patient‐reported outcomes,” and “quality of life.” We carried out a comprehensive search for studies and papers published from 2011 to 2021 that included the use of PRO scales to assess BC patients. Using inclusion and exclusion criteria for literature screening, we retrieved 10,954 papers from four databases, removed duplicates, and screened them, resulting in 237 papers meeting the criteria (Figure 2). The papers were screened against corresponding standards, and scale domains and items were extracted and summarized to form an item pool of 277 PRO items.

We used the item pool and determined that the NCC‐BC‐A scale should include five domains: (1) physiological domain, (2) psychological domain, (3) social domain, (4) therapeutic domain, and (5) overall self‐evaluation domain. BC patients were asked to rate the importance of each item on a 5‐point Likert‐type scale (1 = *not at all*, 2 = *a little bit*, 3 = *somewhat*, 4 = *quite a bit*, 5 = *very much*). A higher score indicated better QOL. The score for each domain was obtained by summing the scores of all items within that domain and dividing by the number of items answered. The total score of the scale was obtained by adding the scores of each domain.

### 2.3 The second phase: Item reduction

This phase focused on conducting a Delphi expert correspondence. Experts from 28 general hospitals in 14 different provincial administrative regions/municipalities were invited to participate in two rounds of correspondence on the content of the scale. The selection criteria for the experts were: (1) at least 5 years of experience in the treatment of breast cancer; (2) an undergraduate degree or above; (3) a job title of intermediate level or above; and (4) voluntary participation in this study. The first round involved 14 experts with an average age of 43.86 years (range: 32–59 years), and an average of 7.71 years of experience (range: 1–18 years). Twelve held associate senior or higher positions, and 13 had master's degrees or higher. The second‐round questionnaire was completed by 31 experts with an average age of 45.07 years (range: 33–58 years), and 6.42 years of experience (range: 1–28 years). Thirty were in associate senior or higher positions, and 29 had master's degrees or higher. All experts worked in departments of medical oncology, breast medicine, or breast surgery. The valid recovery rate for both rounds of questionnaires was 100%. The authority coefficients of the experts were 0.739 and 0.826, and their Kendall harmony coefficients were 0.219 (*p* = 0.008) and 0.098 (*p* < 0.001). Items were selected using a mean importance score of ≥4, full frequency of ≥30%, and coefficient of variation of ≤30%. The entries were adjusted and modified based on the experts' opinions. In the first round of consultation, 63 items were screened out, 9 items were added, and 72 items were retained. In the second round of consultation, 23 items were deleted, resulting in a test scale with 49 items.

A total of 30 patients with advanced BC were selected for cognitive testing to identify any unclear or ambiguous items. Nine additional items were deleted, resulting in a preliminary scale of 40 items.

### 2.4 The third phase: Validation

A small sample of advanced BC patients was presurveyed using the initial beta version of the NCC‐BC‐A scale. Exploratory and confirmatory factor analyses were conducted to assess the validity and reliability of the items and the scale. The beta version of the NCC‐BC‐A scale was then used to conduct a formal survey among a large sample of advanced BC patients to evaluate the scale's reliability, validity, and feasibility.

#### 2.4.1 Participants

Patients with advanced BC were recruited from 63 hospitals in China from October to December 2022 for the preliminary and formal surveys. The inclusion criteria were: (1) aged ≥18 years of age, with no gender limit; (2) pathologically confirmed advanced BC (local recurrence or metastasis); and (3) voluntarily participatation with signed informed consent. The exclusion criteria were: (1) presence of malignant tumors other than BC; (2) mental illness or severe emotional disorders that might affect scoring; (3) cognitive dysfunction; (4) severe heart, brain, lung, liver, or kidney failure; or (5) inability to cooperate in completing the scale or communication difficulties, as evaluated by the investigators.

#### 2.4.2 Questionnaire distribution and data collection

A third party distributed a preliminary survey and a formal survey to patients with advanced BC using QR codes. The patients' sociodemographic information (including gender, age, marital status, education, and employment status) and completed PRO scales were collected.

#### 2.4.3 Statistical analysis

The analysis of items involved the application of various statistical techniques to ensure the robustness and validity of the measurement instrument. The variation coefficient (VC), correlation coefficient, and other exploratory factor analysis (EFA) methods were used to screen items.

The variation coefficient was calculated as the ratio of the standard deviation to the mean. Items with a variation coefficient <0.3 were excluded from further consideration because they did not meet the predefined inclusion criteria. Item‐domain consistency was evaluated by examining the correlation coefficient. Items with a coefficient of <0.20 were subject to further scrutiny. If an item was found to have a correlation coefficient below 0.20 with more than 50% of the other items in its domain, it was considered inconsistent and not suitable for inclusion. The domain correlation coefficient was used to assess the interrelationships among scale domains. Items with coefficients <0.4 were deemed not suitable for inclusion. Cronbach's *α* was also evaluated at item level, domain level, and overall level. Items with coefficients <0.7 were considered unsatisfactory and not suitable for inclusion. In the exploratory factor analysis, items were assessed based on loading thresholds. An item was considered suitable for inclusion if it met the following criteria: (1) its loading on each factor was ≥0.5, and (2) the loading on factors other than its attributed factor was <0.5. The distribution of continuous data was examined using the Shapiro–Wilk test. Normally distributed continuous data were presented as means ± standard deviations (SDs), and skewed data as medians with interquartile ranges (Q1, Q3). Categorical data were summarized as counts and percentages.

Spearman's rank correlation was used to calculate correlation coefficients between variables. Statistical significance was set at a two‐sided *p* < 0.05. All analyses used SPSS 26.0 (IBM Corp).

## 3 RESULTS

### 3.1 Preliminary survey

A total of 200 patients with advanced BC participated in the preliminary survey. All participants were female, with a mean age of 49.1 ± 10.6 years. Most were married (84.5%), had undergraduate education (34.0%), and were unemployed (43.5%) (Table 1). For item #2, “Do you need help from others to eat, dress, wash or go to the toilet?” 86% of patients chose “not at all,” meeting the ceiling effect standard. Therefore, this item was not included in the subsequent analysis.

**Table 1:** Characteristics of the patients participating in the preliminary survey (*n* = 200).

Characteristics	Data
Age (years), mean ± SD	49.1 ± 10.6
Female, *n* (%)	200 (100.0)
Marital status, *n* (%)	
Married	169 (84.5)
Divorced	16 (8.0)
Widowed	12 (6.0)
Unmarried	3 (1.5)
Education, *n* (%)	
Primary school	22 (11.0)
Junior high school	54 (27.0)
Senior high school	53 (26.5)
Undergraduate	68 (34.0)
Graduate student or above	3 (1.5)
Employment status, *n* (%)	
Employed	55 (27.5)
Retirement	58 (29.0)
Unemployed	87 (43.5)

In the preliminary survey, various methods were used to analyze the items, including the variation coefficient, correlation coefficient, internal item consistency, and exploratory factor analysis methods. Items that met the criteria of at least three screening methods were retained. Item #32, “Have you ever had diarrhea?” was deleted because it only met two screening methods. All other items were retained.

Cronbach's *α* was 0.823 for the physiological domain, 0.726 for the psychological domain, 0.710 for the social domain, 0.855 for the therapeutic domain, and 0.879 for the overall self‐evaluation domain (Table 2). Cronbach's *α* for each item is shown in Table S1. The correlation coefficients between items and domains ranged from 0.3 to 0.9 (Table S2). To ensure the remaining 40 items were adequate for factor analysis, the KMO measure of sampling adequacy (0.869) and Bartlett's test of sphericity (*χ* ^2^ = 3993.548, *df* = 666, *p* < 0.001) were assessed and deemed appropriate. The scale was suitable for exploratory factor analysis. The first nine factors were extracted, with a cumulative variance contribution rate was 65.9% (>60%), and eigenvalues of each factor were all >1. The results of the factor analysis after rotation are shown in Table S3.

**Table 2:** Cronbach's *α* for each domain in the preliminary survey and formal survey.

Domains	Cronbach's *α* after deletion of terms	
	Preliminary survey	Formal survey
Physiological domain	0.832	0.882
Psychological domain	0.726	0.640
Social domain	0.710	0.738
Therapeutic domain	0.855	0.869
Overall self‐evaluation domain	0.879	0.827

### 3.2 Formal survey

A total of 588 patients with advanced BC participated in the formal survey. There were 573 women (97.44%) and 15 men (2.56%), with a mean age was 51.59 ± 10.10 years. Most were married (85.03%), had a junior high school education (28.40%), and were retired (39.63%) (Table 3). No floor effect or ceiling effect items were found, suggesting the test items effectively differentiate respondents across the full range of scores without clustering at the lowest or highest ends.

**Table 3:** Characteristics of the patients participating in the formal survey (*n* = 588).

Characteristics	Data
Age (years), mean ± SD	51.59 ± 10.10
Female, *n* (%)	573 (97.45)
Male, *n* (%)	15 (2.55)
Marital status, *n* (%)	
Married	500 (85.03)
Divorced	37 (6.29)
Widowed	34 (5.78)
Unmarried	17 (2.89)
Education, *n* (%)	
Primary school	121 (20.58)
Junior high school	167 (28.40)
Senior high school	148 (25.17)
Undergraduate	143 (24.32)
Graduate student or above	9 (1.53)
Employment status, *n* (%)	
On‐the‐job training	1 (0.17)
Employed	205 (34.86)
Retirement	233 (39.63)
Unemployed	149 (25.34)

The same screening method was applied as in the preliminary survey. Items #14, #16, and #21 were removed because they only met one screening method. After discussion among experts, items #14, #16, and #21 were changed to “Do you feel irritable, depressed, or that you have lost hope because of illness?” “Does the illness affect your family responsibilities (such as housework, family income)?” and “Do you often feel tired when you are doing leisure activities?” This resulted in a final scale comprising 38 items (see in APPENDIX).

To assess the test–retest reliability, 55 participants were randomly selected from the patients with advanced BC who participated in the first formal survey, and the survey was conducted again after 2 weeks. The test–retest reliability was 0.9041 (*p* = 0.001).

The Cronbach's *α* coefficient was 0.925 for the overall scale, 0.882 for the physiological domain, 0.640 for the psychological domain, 0.738 for the social domain, 0.869 for the therapeutic domain, and 0.827 for the overall self‐evaluation domain (Table 2). Cronbach's *α* for each item is shown in Table S1. The correlation coefficients between items and domains ranged from 0.2 to 0.9 (Table S4).

**Figure 3:**
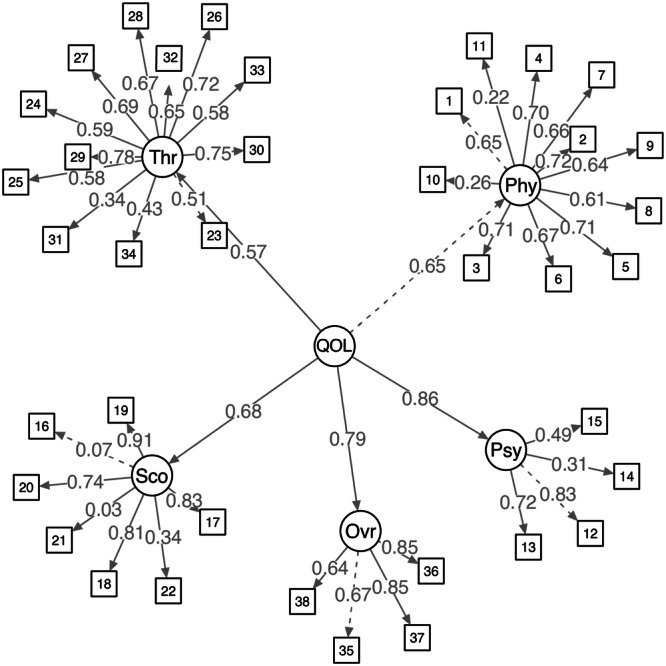
Final confirmatory factor analysis model. Fit indices: *χ* ^2^ = 3402.96.38, *p* < 0.001, comparative fit index = 0.74, root mean square error of approximation = 0.08, standardized root mean squared residual = 0.12. Ovr, Overall self‐evaluation domain; Phy, Physiological domain; Psy, Psychological domain; Sco, Social domain; Thr, Therapeutic domain; QOL, quality of life.

We also defined a QOL factor to incorporate loadings from each of the five sections and 38 items. In the initial model estimation, moderate to strong standardized factor loadings ranging from 0.57 to 0.86 were observed, indicative of a well‐fitted model with robust global fit indices (Figure 3). The therapeutic domain displayed the lowest loading, while the psychological domain had the highest. This finding suggests potential areas for future research focusing on enhancing the structural elements of the therapeutic domain and the PRO. The inclusion of additional patient data in subsequent analyses could provide valuable insights and further refine the understanding of these relationships.

### 3.3 Fiducial value setting of the scale

Based on the 588 surveys from patients with advanced BC in the formal survey phase, the statistician set the fiducial values of each domain as 3.59 for the physiological domain, 3.16 for the psychological domain, 3.38 for the social domain, 3.63 for the therapeutic domain, and 3.14 for the overall self‐evaluation domain. The fiducial values can assist in the PRO assessment for BC patients, with specific scores and standard settings are shown in Table S5.

## 4 DISCUSSION

Several PRO tools are available for BC patients, such as the EORTC QLQ‐C30 and FACT‐B, but these were developed and validated in Western countries. This study aimed to develop and validate the reliability and validity of a new PRO scale, NCC‐BC‐A scale, to assess treatment‐related QOL among Chinese BC patients. The results suggest that the NCC‐BC‐A scale was successfully validated. This scale considers the disease characteristics and cultural differences of Chinese patients with BC, making it suitable for a comprehensive and accurate assessment of the QOL.

The NCC‐BC‐A scale was developed following the PRO development process emphasized by the CDE of the National Medical Products Administration in China. The formal development and validation process included establishing an item pool, conducting two rounds of Delphi expert consultation, performing cognitive testing with patients, and carrying out preliminary and formal surveys. The results showed relatively stable consistency and coordination, suggesting that this method is feasible and the results credible. The final scale contains 38 items with high test–retest reliability.

In advanced BC, the endpoints required for supporting drug approval usually include overall survival (OS), progression‐free survival (PFS), and objective response rate (ORR) [19]. Unlike several other types of aggressive and lethal cancer, such as pancreatic cancer, the median OS of patients with BC is relatively long, and it is common for patients to receive multiple lines of treatments and experience prolonged survival in the metastatic setting [1, 20]. Therefore, considering the QOL of the patients with advanced BC is crucial, as poor QOL can influence or confound OS benefits [21]. Achieving a longer OS without worsening or improved QOL is the primary therapeutic goal for these patients [22]. It is important to consider patients' subjective feelings, as reflected in PROs, which can reflect the severity of symptoms and impact of treatment on QOL and survival.

All systemic treatments for BC induce adverse drug reactions of varying natures and variable intensities, depending on the treatment. These adverse events significantly impact patients' QOL because of the long duration of treatment [23]. Existing scales, such as the EORTC QLQ‐C30, EORTC QLQ‐BR23 [13, 14, 15], and FACT‐B [16], can assess the PRO of BC patients, but these questionnaires were designed and validated in Western populations, and the EORTC QLQ‐C30 is not specific to BC. The QLQ‐CCC questionnaire was developed in 1997 to evaluate the QOL of Chinese cancer patients receiving chemobiotherapy, but it does not comprehensively assess adverse events during the treatment [24]. It was developed before targeted therapies and immunotherapy. The NCC‐BC‐A scale focuses on adverse reactions caused by contemporary treatment for BC. For example, in the formal survery, 62.25% of patients reported experiencing “hair loss due to treatment,” while 55.79% of patients reported feeling that their “immunity is reduced.” These findings indicate that most BC patients have adverse reactions, making the inclusion of these items in the scale necessary.

In recent years, several targeted therapies for the treatment of BC, such as palbociclib, abemaciclib, everolimus, trastuzumab emtansinem, and trastuzumab deruxtecan, have been approved for marketing in China. These agents have specific adverse reaction patterns that must be considered [25, 26, 27, 28]. Existing international scales currently used in clinical practice, such as the EORTC QLQ‐C30 and FACT‐B, were designed when there were few targeted therapies for BC. However, several targeted agents, such as CDK4/6 inhibitors, are now widely used in current clinical practice. Some novel targeted therapies are taken orally every day at home. Therefore, PRO tools are particularly important since physicians may only be able to evaluate patients sporadically during treatment. It is necessary to optimize existing scale for targeted therapies. The new scale specifically targets patients with advanced BC receiving systemic treatment, improving its sensitivity. This makes it different from the QLICP‐BR V2.0 scale, which has been validated in Chinese patients with BC regardless of their disease phase [29], and only considers general adverse reactions rather than specific adverse reactions.

Despite its benefits, this study also has limitations. First, during the presurvey and formal survey phases, no comparisons were made with previous validated scales, such as the EORTC QLQ‐C30 scale and FACT‐B scale. Consequently, the correlative validity between the new scale and previously validated scales could not be determined. Additionally, the inclusion and exclusion criteria restricted the recruitment to patients with advanced BC for preliminary and formal surveys, excluding those with early BC, which may limit the scale's generalizability.

## 5 CONCLUSION

In conclusion, the development of the NCC‐BC‐A scale involved a comprehensive literature review, establishment of an item pool, and rigorous validation processes. The NCC‐BC‐A scale fully considers the characteristics of current treatment for BC patients and the realistic therapeutic situation in China. It may help clinicians to focus on the QOL of advanced BC patients and optimize the management system for treating BC in China.

## AUTHOR CONTRIBUTIONS

**Fei Ma**: Conceptualization (lead); funding acquisition (lead); investigation (lead); project administration (lead); resources (lead); supervision (lead); writing—review & editing (lead); **Xiaoyan Yan**: Formal analysis (lead); investigation (equal); methodology (lead); project administration (lead); software (lead); writing—review & editing (equal); **Xiuwen Guan**: Data curation (equal); formal analysis (equal); investigation (equal); project administration (equal); resources (equal); writing—original draft (lead); writing—review & editing (equal); **Tianmou Liu**: Data curation (equal); formal analysis (equal); methodology (lead); software (lead); writing—original draft (equal); writing—review & editing (equal).

## CONFLICT OF INTEREST STATEMENT

Professor Fei Ma is the member of the *Cancer Innovation* Editorial Board. To minimize bias, he was excluded from all editorial decision‐making related to the acceptance of this article for publication. The remaining authors declare no conflict of interest.

## ETHICS STATEMENT

This study was approved by the Ethics Committee of National Cancer Center/Cancer Hospital, Chinese Academy of Medical Sciences and Peking Union Medical College (approval number 22/245‐3447).

## INFORMED CONSENT

All participating doctors, nurses and patients provided written informed consent.

## Supporting information

Supporting information.

## Data Availability

The data sets analyzed during current study are available from the corresponding author upon reasonable request.

## THE NCC‐BC‐A SCALE.

### 1

Patients sometimes report that they have the following symptoms or problems. Please indicate the extent to which you have had these symptoms or problems during the past week.

**Table:**

Item	Degree				
Physiological domain	Not at all	A little bit	Some‐what	Quite a bit	Very much
1. Do you need assistance with performing your usual activities outside?					
2. Have you ever had pain?					
3. Do you have significant pain in certain parts of your body?					
4. Have you ever felt nausea or vomiting?					
5. Have you eaten less?					
6. Have you had trouble sleeping?					
7. Have you ever felt tired?					
8. Do you have memory loss?					
9. Do you have trouble remembering where to put things? For example, do you have trouble remembering where you put your keys or wallet?					
10. Did you have sex?	Yes (to 11)	No (to 12)			
11. Did you have a good sex life with your partner?					
Psychological domain	Not at all	A little bit	Some‐what	Quite a bit	Very much
12. Are you becoming more confident in the fight against your illness?					
13. Have you accepted your illness?					
14. Do you feel agitated, depressed or hopeless because of your illness?					
15. Do you feel fulfilling in your work (include work at home)?					
Social domain	Not at all	A little bit	Some‐what	Quite a bit	Very much
16. Has the illness affected your ability to take on family responsibilities? (such as undertaking housework, family income, etc.)					
17. Did you get support from your family during your treatment?					
18. Do you feel close to your partner (or the person who is your main support)?					
19. Have you been able to get along well with your family even after your illness?					
20. Have you got encouragement from friends?					
21. Do you often feel tired when doing leisure activities?					
22. Have you been satisfied with your body?					
Therapeutic domain	Not at all	A little bit	Some‐what	Quite a bit	Very much
23. Has the treatment of illness increased your discomfort?					
24. Have you ever felt a decrease in immunity?					
25. Have you ever felt lymphedema?					
26. Have you ever felt dizzy?					
27. Have you ever felt panic, palpitations, rapid heartbeat?					
28. Have you ever felt numbness in your hands and feet?					
29. Have you ever felt unresponsive in your hands and feet?					
30. Have you ever felt that your hands and feet are not sensitive to heat and cold?					
31. Have you ever had hair loss due to treatment?					
32. Have you ever had redness, swelling, or peeling of the skin on your hands and feet?					
33. Have you ever been constipated?					
Other domain	Not at all	A little bit	Some‐what	Quite a bit	Very much
34. Has your physical condition or medical treatment caused you financial difficulties?					
Overall self‐evaluation domain					
35. Are you satisfied with your current treatment?	Not at all	A little bit	Some‐what	Quite a bit	Very much
36. How good or bad do you think your physical condition is today?	Very poor	Quite poor	General	Quite good	Very good
37. How would you rate your overall physical condition during the past week?	Very poor	Quite poor	General	Quite good	Very good
38. Has your health gotten worse in general now compared to 1 year ago?	Very poor	Quite poor	General	Quite good	Very good

## 乳腺癌患者报告结局量表 (NCC‐BC‐A) (Chinese Edition)

### 1

患者有时会报告以下症状或问题。请回忆您在过去一周中出现这些症状或问题的程度。

**Table:**

条目	程度				
生理维度	一点也不	有一点	有些	相当	非常
1. 您平时日常外出活动需要人协助吗？					
2. 您有过疼痛吗？					
3. 您身体的某些部位有明显疼痛感吗？					
4. 您曾感受到恶心想吐吗？					
5. 您的食量有减少吗？					
6. 您会感觉入睡困难吗？					
7. 您曾感觉到疲乏吗？					
8. 您有记忆力减退现象吗？					
9. 您在记起把东西放到哪里方面有困难吗？例如, 您记不起把钥匙或钱包之类的东西放到哪里了					
10. 您有性生活吗？	有 (到11题)	无 (到12题)			
11. 您与伴侣的性生活和谐吗？					
心理维度	一点也不	有一点	有些	相当	非常
12. 您在与疾病的抗争中, 愈来愈有信心吗？					
13. 您能面对自己的疾病了吗？					
14. 您因疾病感受到烦躁不安、情绪低落或失去希望吗？					
15. 您的工作 (包括家务) 令您有成就感吗？					
社会维度	一点也不	有一点	有些	相当	非常
16. 生病是否影响了您承担家庭责任？(比如承担家务、家庭收入等)					
17. 您在治疗过程中, 得到了家人的支持吗？					
18. 您与自己的配偶 (或给您主要支持的人) 关系很密切吗？					
19. 您患病后, 依然能与家人很好地相处吗？					
20. 您得到过朋友的言语鼓励吗？					
21. 您进行休闲活动时经常感到疲惫吗？					
22. 您对自己的身体满意吗？					
治疗维度	一点也不	有一点	有些	相当	非常
23. 疾病的治疗增加了您的不适感吗？					
24. 您曾感觉到免疫力下降吗？					
25. 您曾感觉到淋巴水肿吗？					
26. 您曾感觉到头晕吗？					
27. 您曾感觉到心慌、心悸、心跳加速吗？					
28. 您曾感觉到手脚麻木吗？					
29. 您曾感觉到手脚反应迟钝吗？					
30. 您曾感觉到手脚对冷热不敏感吗？					
31. 您曾有因为治疗引起脱发吗？					
32. 您的手脚出现过红肿、脱皮吗？					
33. 您曾有便秘吗？					
其他维度	一点也不	有一点	有些	相当	非常
34. 您的身体状况或治疗过程, 造成了您的经济困难吗？					
总体自我评价维度					
35. 您对现在的治疗状况满意吗？	一点也不	有一点	有些	相当	非常
36. 您认为自己今天的健康状况好坏程度如何？	很差	比较差	一般	比较好	很好
37. 您如何评定过去一周中您的整体健康状况？	很差	比较差	一般	比较好	很好
38. 您现在的健康状况与一年前的健康状况相比变差了吗？	很差	比较差	一般	比较好	很好
