# Supplementary material for: Development and validation of the NCC‐BC‐A scale to assess patient‐reported outcomes for breast cancer patients in China
Source: Cancer Innov. 2024 Oct 18;3(6):e141. doi: 10.1002/cai2.141 (PMC11487329; doi:10.1002/cai2.141)
Supplement: Supplementary file 1 — Supporting information. [file CAI2-3-e141-s001.docx]

**Development and validation of the NCC-BC-A scale to assess patient-reported outcomes for breast cancer patients in China**

**Supplementary Table 1.** Cronbach’s alpha for each item in the preliminary survey and formal survey

| Items | Cronbach's alpha after deletion of terms | |
| --- | --- | --- |
|  | Preliminary survey | Formal survey |
| 1 | 0.737 | 0.872 |
| 2 | − | 0.867 |
| 3 | 0.729 | 0.868 |
| 4 | 0.729 | 0.867 |
| 5 | 0.728 | 0.866 |
| 6 | 0.731 | 0.869 |
| 7 | 0.748 | 0.869 |
| 8 | 0.734 | 0.872 |
| 9 | 0.731 | 0.870 |
| 10 | 0.743 | − |
| 11 | 0.742 | − |
| 12 | − | 0.403 |
| 13 | − | 0.466 |
| 14 | 0.733 | 0.726 |
| 15 | 0.750 | 0.600 |
| 16 | 0.783 | 0.770 |
| 17 | 0.767 | 0.656 |
| 18 | 0.735 | 0.666 |
| 19 | 0.715 | 0.632 |
| 20 | 0.703 | 0.668 |
| 21 | 0.704 | 0.775 |
| 22 | 0.728 | 0.731 |
| 23 | 0.733 | 0.862 |
| 24 | 0.715 | 0.856 |
| 25 | 0.740 | 0.860 |
| 26 | 0.736 | 0.852 |
| 27 | 0.748 | 0.853 |
| 28 | 0.744 | 0.853 |
| 29 | 0.740 | 0.848 |
| 30 | 0.732 | 0.850 |
| 31 | 0.730 | 0.878 |
| 32 | 0.735 | 0.854 |
| 33 | 0.738 | 0.860 |
| 34 | 0.741 | − |
| 35 | 0.737 | 0.828 |
| 36 | − | 0.742 |
| 37 | 0.799 | 0.743 |
| 38 | 0.786 | 0.815 |
| 39 | 0.791 | − |
| 40 | 0.786 | − |

**Supplementary Table 2.** Correlation coefficients between items and domains in the preliminary survey

| Items | physiological domain | psychological domain | social domain | treatment domain | overall self-evaluation domain |
| --- | --- | --- | --- | --- | --- |
| 1 | 0.582 | 0.288 | 0.327 | 0.363 | 0.409 |
| 3 | 0.651 | 0.293 | 0.321 | 0.423 | 0.382 |
| 4 | 0.656 | 0.259 | 0.316 | 0.356 | 0.350 |
| 5 | 0.640 | 0.313 | 0.257 | 0.468 | 0.445 |
| 6 | 0.634 | 0.340 | 0.303 | 0.421 | 0.476 |
| 7 | 0.385 | 0.238 | 0.265 | 0.253 | 0.221 |
| 8 | 0.606 | 0.285 | 0.189 | 0.357 | 0.263 |
| 9 | 0.701 | 0.432 | 0.350 | 0.554 | 0.543 |
| 10 | 0.520 | 0.287 | 0.222 | 0.461 | 0.291 |
| 11 | 0.510 | 0.317 | 0.269 | 0.424 | 0.252 |
| 14 | 0.370 | 0.822 | 0.517 | 0.242 | 0.598 |
| 15 | 0.352 | 0.776 | 0.442 | 0.210 | 0.456 |
| 16 | 0.450 | 0.613 | 0.393 | 0.398 | 0.370 |
| 17 | 0.299 | 0.680 | 0.429 | 0.220 | 0.387 |
| 18 | 0.346 | 0.269 | 0.493 | 0.369 | 0.327 |
| 19 | 0.179 | 0.340 | 0.672 | 0.154 | 0.268 |
| 20 | 0.180 | 0.379 | 0.694 | 0.193 | 0.249 |
| 21 | 0.249 | 0.472 | 0.732 | 0.162 | 0.292 |
| 22 | 0.017 | 0.222 | 0.513 | -0.007 | 0.120 |
| 23 | 0.391 | 0.269 | 0.466 | 0.403 | 0.392 |
| 24 | 0.405 | 0.593 | 0.596 | 0.321 | 0.606 |
| 25 | 0.531 | 0.392 | 0.432 | 0.631 | 0.556 |
| 26 | 0.535 | 0.350 | 0.341 | 0.689 | 0.494 |
| 27 | 0.271 | 0.217 | 0.267 | 0.430 | 0.170 |
| 28 | 0.418 | 0.262 | 0.197 | 0.541 | 0.314 |
| 29 | 0.495 | 0.242 | 0.186 | 0.564 | 0.333 |
| 30 | 0.471 | 0.209 | 0.255 | 0.712 | 0.307 |
| 31 | 0.500 | 0.307 | 0.359 | 0.769 | 0.363 |
| 32 | 0.379 | 0.184 | 0.312 | 0.659 | 0.294 |
| 33 | 0.269 | 0.120 | 0.128 | 0.580 | 0.220 |
| 34 | 0.310 | 0.009 | 0.098 | 0.533 | 0.136 |
| 35 | 0.450 | 0.210 | 0.198 | 0.632 | 0.348 |
| 36 | 0.353 | 0.298 | 0.349 | 0.391 | 0.303 |
| 37 | 0.391 | 0.481 | 0.415 | 0.316 | 0.797 |
| 38 | 0.541 | 0.560 | 0.506 | 0.492 | 0.892 |
| 39 | 0.572 | 0.551 | 0.510 | 0.477 | 0.887 |
| 40 | 0.551 | 0.515 | 0.459 | 0.514 | 0.848 |

**Supplementary Table 3.** The exploratory factor analysis in the preliminary survey

| Items | Factor 1 | Factor 2 | Factor 3 | Factor 4 | Factor 5 | Factor 6 | Factor 7 | Factor 8 | Factor 9 |
| --- | --- | --- | --- | --- | --- | --- | --- | --- | --- |
| 1 | 0.219 | 0.331 | −0.044 | 0.078 | 0.329 | 0.441 | −0.111 | 0.340 | −0.115 |
| 3 | 0.210 | 0.188 | 0.076 | 0.028 | 0.799 | 0.211 | 0.094 | 0.184 | 0.139 |
| 4 | 0.226 | 0.106 | 0.071 | 0.035 | 0.829 | 0.193 | 0.031 | 0.175 | 0.105 |
| 5 | 0.257 | 0.157 | −0.103 | 0.106 | 0.350 | 0.661 | 0.144 | 0.107 | 0.103 |
| 6 | 0.340 | 0.149 | −0.114 | 0.042 | 0.257 | 0.658 | 0.168 | 0.193 | 0.025 |
| 7 | 0.030 | 0.091 | 0.174 | −0.013 | 0.034 | 0.716 | −0.010 | 0.101 | 0.029 |
| 8 | 0.078 | −0.040 | 0.029 | 0.465 | 0.578 | 0.115 | 0.198 | −0.119 | 0.005 |
| 9 | 0.428 | 0.247 | −0.023 | 0.435 | 0.199 | 0.246 | 0.259 | 0.064 | −0.055 |
| 10 | 0.136 | 0.179 | 0.089 | 0.800 | 0.007 | −0.048 | 0.158 | 0.003 | 0.086 |
| 11 | 0.062 | 0.183 | 0.118 | 0.737 | 0.074 | 0.013 | −0.013 | 0.165 | 0.156 |
| 14 | 0.707 | −0.148 | 0.276 | 0.206 | −0.037 | 0.146 | 0.010 | 0.013 | 0.185 |
| 15 | 0.561 | −0.078 | 0.298 | 0.150 | 0.087 | 0.097 | −0.102 | 0.013 | 0.301 |
| 16 | 0.321 | 0.147 | 0.134 | 0.535 | 0.084 | 0.090 | 0.024 | 0.306 | −0.002 |
| 17 | 0.464 | −0.093 | 0.265 | 0.124 | 0.043 | 0.123 | 0.005 | 0.284 | −0.051 |
| 18 | 0.145 | −0.021 | 0.049 | 0.022 | 0.110 | 0.204 | 0.190 | 0.696 | 0.171 |
| 19 | 0.195 | 0.110 | 0.766 | 0.014 | 0.074 | −0.032 | −0.080 | −0.006 | 0.108 |
| 20 | 0.108 | 0.063 | 0.802 | 0.024 | 0.068 | 0.021 | 0.177 | 0.047 | −0.079 |
| 21 | 0.233 | 0.116 | 0.813 | 0.100 | 0.120 | −0.005 | −0.065 | 0.090 | −0.094 |
| 22 | 0.068 | −0.047 | 0.682 | 0.067 | −0.126 | 0.025 | −0.073 | −0.069 | 0.061 |
| 23 | 0.304 | 0.202 | −0.080 | 0.161 | 0.128 | 0.088 | 0.081 | 0.627 | −0.044 |
| 24 | 0.724 | 0.089 | 0.103 | 0.106 | 0.092 | 0.046 | −0.079 | 0.250 | −0.027 |
| 25 | 0.475 | 0.326 | −0.002 | 0.157 | 0.083 | 0.161 | 0.358 | 0.340 | −0.190 |
| 26 | 0.381 | 0.236 | −0.109 | 0.248 | 0.182 | 0.157 | 0.440 | 0.288 | −0.006 |
| 27 | 0.087 | 0.329 | 0.086 | 0.075 | 0.232 | −0.136 | −0.076 | 0.285 | 0.641 |
| 28 | 0.207 | 0.088 | −0.061 | 0.381 | 0.182 | 0.203 | 0.149 | −0.076 | 0.577 |
| 29 | 0.241 | 0.397 | −0.080 | 0.436 | 0.058 | 0.430 | 0.036 | −0.054 | 0.189 |
| 30 | 0.135 | 0.738 | 0.098 | 0.274 | 0.175 | 0.071 | 0.158 | −0.072 | 0.081 |
| 31 | 0.169 | 0.726 | 0.111 | 0.267 | 0.161 | 0.153 | 0.168 | 0.149 | 0.191 |
| 32 | 0.100 | 0.784 | 0.090 | 0.086 | 0.019 | 0.205 | 0.167 | 0.136 | 0.097 |
| 33 | 0.120 | 0.163 | −0.031 | 0.025 | 0.034 | 0.038 | 0.773 | 0.065 | 0.026 |
| 34 | −0.053 | 0.388 | −0.073 | 0.107 | −0.111 | 0.268 | 0.384 | 0.009 | 0.497 |
| 35 | 0.190 | 0.270 | −0.095 | 0.230 | 0.356 | −0.050 | 0.546 | 0.129 | 0.057 |
| 36 | 0.054 | 0.001 | 0.303 | 0.172 | 0.071 | 0.170 | 0.445 | 0.385 | 0.258 |
| 37 | 0.744 | 0.115 | 0.100 | 0.017 | 0.022 | 0.110 | 0.114 | −0.076 | 0.031 |
| 38 | 0.767 | 0.244 | 0.107 | 0.031 | 0.222 | 0.017 | 0.153 | 0.171 | 0.134 |
| 39 | 0.761 | 0.222 | 0.123 | 0.095 | 0.240 | 0.045 | 0.141 | 0.142 | 0.022 |
| 40 | 0.730 | 0.144 | 0.026 | 0.122 | 0.164 | 0.158 | 0.237 | 0.105 | −0.040 |

**Supplementary Table 4.** Correlation coefficients between items and domains in the formal survey

| Items | physiological domain | psychological domain | social domain | treatment domain | overall self-evaluation domain |
| --- | --- | --- | --- | --- | --- |
| 1 | 0.6831 | 0.3758 | 0.4042 | 0.3775 | 0.2929 |
| 3 | 0.7350 | 0.3289 | 0.3376 | 0.4204 | 0.2739 |
| 4 | 0.7191 | 0.3725 | 0.3428 | 0.408 | 0.3304 |
| 5 | 0.7334 | 0.3800 | 0.3467 | 0.4822 | 0.2799 |
| 6 | 0.7509 | 0.3927 | 0.3667 | 0.4685 | 0.2887 |
| 7 | 0.7242 | 0.3287 | 0.3340 | 0.4742 | 0.3142 |
| 8 | 0.7180 | 0.2788 | 0.2676 | 0.5257 | 0.2804 |
| 9 | 0.6897 | 0.2620 | 0.2965 | 0.5040 | 0.2537 |
| 12 | 0.3653 | 0.8319 | 0.5702 | 0.2722 | 0.5842 |
| 13 | 0.2985 | 0.7818 | 0.5076 | 0.1911 | 0.4505 |
| 14 | 0.5376 | 0.4656 | 0.3286 | 0.5116 | 0.2241 |
| 15 | 0.1130 | 0.6641 | 0.4068 | 0.1196 | 0.4013 |
| 16 | 0.3544 | 0.1583 | 0.3551 | 0.3470 | 0.1859 |
| 17 | 0.2741 | 0.5140 | 0.7785 | 0.1962 | 0.4664 |
| 18 | 0.2002 | 0.4699 | 0.7559 | 0.1886 | 0.3273 |
| 19 | 0.3041 | 0.5516 | 0.8411 | 0.2053 | 0.4345 |
| 20 | 0.2565 | 0.4326 | 0.7441 | 0.1955 | 0.4210 |
| 21 | 0.3625 | 0.2338 | 0.2957 | 0.4075 | 0.1463 |
| 22 | 0.3370 | 0.4883 | 0.5299 | 0.2599 | 0.5443 |
| 23 | 0.3944 | 0.2591 | 0.2445 | 0.5843 | 0.2432 |
| 24 | 0.4203 | 0.2424 | 0.2302 | 0.6617 | 0.2252 |
| 25 | 0.4345 | 0.3385 | 0.3478 | 0.6215 | 0.1871 |
| 26 | 0.5780 | 0.3081 | 0.3247 | 0.7220 | 0.2806 |
| 27 | 0.5214 | 0.2463 | 0.2899 | 0.6986 | 0.2754 |
| 28 | 0.3862 | 0.1414 | 0.2365 | 0.7077 | 0.1890 |
| 29 | 0.4981 | 0.3014 | 0.3444 | 0.7646 | 0.2824 |
| 30 | 0.5184 | 0.3194 | 0.3800 | 0.7334 | 0.3227 |
| 31 | 0.1945 | 0.0905 | 0.0195 | 0.4940 | 0.0858 |
| 32 | 0.4274 | 0.3046 | 0.2949 | 0.6945 | 0.2902 |
| 33 | 0.4004 | 0.3044 | 0.3011 | 0.629 | 0.2581 |
| 34 | 0.3385 | 0.2033 | 0.2138 | 0.4377 | 0.2245 |
| 35 | 0.2569 | 0.5932 | 0.5366 | 0.188 | 0.7891 |
| 36 | 0.3786 | 0.5198 | 0.5097 | 0.3324 | 0.8610 |
| 37 | 0.4217 | 0.4768 | 0.5127 | 0.3636 | 0.8600 |
| 38 | 0.2868 | 0.3802 | 0.3362 | 0.3033 | 0.7600 |

**Supplementary Table 5.** Fiducial value setting of each domain

| Domain name | Content | Fiducial value | Significance |
| --- | --- | --- | --- |
| Physiological domain | Movement ability, pain, nausea and vomiting, loss of appetite, difficulty in falling asleep, fatigue, memory loss, sexual function, and other symptoms and physiological functions | 3.59 | >3.59: Patients’ own states are strong.  ≤3.59: Patients’ own states are weak. |
| Psychological domain | Attitude towards disease, psychological state such as anxiety, depression | 3.16 | >3.16: Patients have a more positive and optimistic attitude.  ≤3.16: Patients may be more depressed, anxious, etc. |
| Social domain | Patients communicate with their families, friends, and their social activities | 3.38 | >3.38: Patients’ families and friends give more confidence to these patients.  ≤3.38: Patients’ families and friends need to care more about these patients. |
| Treatment domain | Such as anemia, decreased neural cells, heart toxicity, peripheral neuropathy, hand-foot syndrome, constipation, etc. | 3.63 | >3.63: Small side effects of drugs.  ≤3.63: Large side effects of drugs. |
| Other domain | Economic difficulties caused by physical condition or treatment process | 3.13 | >3.13: Disease treatment has not yet caused economic difficulties.  ≤3.13: Disease treatment has caused economic difficulties. |
| Overall self-evaluation domain | Patients evaluate their overall health status | 3.14 | >3.14: Patients feel pretty good based on their situations.  ≤3.14: Patients feel that their mental states, bodies, and other aspects are not very good. |

Appendix: The NCC-BC-A scale

Patients sometimes report that they have the following symptoms or problems. Please indicate the extent to which you have had these symptoms or problems during the past week.

| Physical domain | Not at  all | A little bit | Some-  what | Quite a bit | Very much |
| --- | --- | --- | --- | --- | --- |
| 1.Do you need assistance with performing your usual activities outside? |  |  |  |  |  |
| 2.Have you ever had pain? |  |  |  |  |  |
| 3.Do you have significant pain in certain parts of your body? |  |  |  |  |  |
| 4.Have you ever felt nausea or vomiting? |  |  |  |  |  |
| 5.Have you eaten less? |  |  |  |  |  |
| 6.Have you had trouble sleeping? |  |  |  |  |  |
| 7.Have you ever felt tired? |  |  |  |  |  |
| 8.Do you have memory loss? |  |  |  |  |  |
| 9.Do you have trouble remembering where to put things? For example, do you have trouble remembering where you put your keys or wallet? |  |  |  |  |  |
| 10.Did you have sex? | Yes (to 11) | No (to 12) |  |  |  |
| 11.Did you have a good sex life with your partner? |  |  |  |  |  |
| Psychological domain | Not at  all | A little bit | Some-  what | Quite a bit | Very much |
| 12.Are you becoming more confident in the fight against your illness? |  |  |  |  |  |
| 13. Have you accepted your illness? |  |  |  |  |  |
| 14. Do you feel agitated, depressed or hopeless because of your illness? |  |  |  |  |  |
| 15. Do you feel fulfilling in your work (include work at home)? |  |  |  |  |  |
| Social domain | Not at  all | A little bit | Some-  what | Quite a bit | Very much |
| 16.Has the illness affected your ability to take on family responsibilities?（ such as undertaking housework, family income, etc.） |  |  |  |  |  |
| 17. Did you get support from your family during your treatment? |  |  |  |  |  |
| 18. Do you feel close to your partner (or the person who is your main support)? |  |  |  |  |  |
| 19. Have you been able to get along well with your family even after your illness? |  |  |  |  |  |
| 20.Have you got encouragement from friends? |  |  |  |  |  |
| 21. Do you often feel tired when doing leisure activities? |  |  |  |  |  |
| 22. Have you been satisfied with your body? |  |  |  |  |  |
| Therapeutic domain | Not at  all | A little bit | Some-  what | Quite a bit | Very much |
| 23.Has the treatment of illness increased your discomfort? |  |  |  |  |  |
| 24.Have you ever felt a decrease in immunity? |  |  |  |  |  |
| 25.Have you ever felt lymphedema? |  |  |  |  |  |
| 26.Have you ever felt dizzy? |  |  |  |  |  |
| 27.Have you ever felt panic, palpitations, rapid heartbeat? |  |  |  |  |  |
| 28. Have you ever felt numbness in your hands and feet? |  |  |  |  |  |
| 29. Have you ever felt unresponsive in your hands and feet? |  |  |  |  |  |
| 30. Have you ever felt that your hands and feet are not sensitive to heat and cold? |  |  |  |  |  |
| 31. Have you ever had hair loss due to treatment? |  |  |  |  |  |
| 32. Have you ever had redness, swelling, or peeling of the skin on your hands and feet? |  |  |  |  |  |
| 33. Have you ever been constipated? |  |  |  |  |  |
| Other domain | Not at  all | A little bit | Some-  what | Quite a bit | Very much |
| 34.Has your physical condition or medical treatment caused you financial difficulties? |  |  |  |  |  |
| Overall self-evaluation domain | Not at  all | A little bit | Some-  what | Quite a bit | Very much |
| 35. Are you satisfied with your current treatment? |  |  |  |  |  |
| 36.How good or bad do you think your physical condition is today? | Very poor | Quite poor | General | Quite good | Very good |
| 37. How would you rate your overall physical condition during the past week? | Very poor | Quite poor | General | Quite good | Very good |
| 38. Has your health gotten worse in general now compared to one year ago? | Very poor | Quite poor | General | Quite good | Very good |

Appendix: The NCC-BC-A scale

患者有时会报告以下症状或问题。请回忆您在过去一周中出现这些症状或问题的程度。

| 生理维度 | 一点也不 | 有一点 | 有些 | 相当 | 非常 |
| --- | --- | --- | --- | --- | --- |
| 1. 您平时日常外出活动需要人协助吗？ |  |  |  |  |  |
| 2.您有过疼痛吗？ |  |  |  |  |  |
| 3.您身体的某些部位有明显疼痛感吗？ |  |  |  |  |  |
| 4.您曾感受到恶心想吐吗？ |  |  |  |  |  |
| 5.您的食量有减少吗？ |  |  |  |  |  |
| 6.您会感觉入睡困难吗？ |  |  |  |  |  |
| 7.您曾感觉到疲乏吗？ |  |  |  |  |  |
| 8.您有记忆力减退现象吗？ |  |  |  |  |  |
| 9.您在记起把东西放到哪里方面有困难吗？例如，您记不起把钥匙或钱包之类的东西放到哪里了 |  |  |  |  |  |
| 10.您有性生活吗? | 有（到11题） | 无（到12题） |  |  |  |
| 11.您与伴侣的性生活和谐吗？ |  |  |  |  |  |
| 心理维度 | 一点也不 | 有一点 | 有些 | 相当 | 非常 |
| 12.您在与疾病的抗争中，愈来愈有信心吗？ |  |  |  |  |  |
| 13.您能面对自己的疾病了吗？ |  |  |  |  |  |
| 14.您因疾病感受到烦躁不安、情绪低落或失去希望吗？ |  |  |  |  |  |
| 15.您的工作（包括家务）令您有成就感吗？ |  |  |  |  |  |
| 社会维度 | 一点也不 | 有一点 | 有些 | 相当 | 非常 |
| 16.生病是否影响了您承担家庭责任？(比如承担家务，家庭收入等) |  |  |  |  |  |
| 17.您在治疗过程中，得到了家人的支持吗？ |  |  |  |  |  |
| 18.您与自己的配偶（或给您主要支持的人）关系很密切吗？ |  |  |  |  |  |
| 19.您患病后，依然能与家人很好地相处吗？ |  |  |  |  |  |
| 20.您得到过朋友的言语鼓励吗？ |  |  |  |  |  |
| 21.您进行休闲活动时经常感到疲惫吗？ |  |  |  |  |  |
| 22.您对自己的身体满意吗？ |  |  |  |  |  |
| 治疗维度 | 一点也不 | 有一点 | 有些 | 相当 | 非常 |
| 23.疾病的治疗增加了您的不适感吗？ |  |  |  |  |  |
| 24.您曾感觉到免疫力下降吗？ |  |  |  |  |  |
| 25.您曾感觉到淋巴水肿吗？ |  |  |  |  |  |
| 26.您曾感觉到头晕吗？ |  |  |  |  |  |
| 27.您曾感觉到心慌、心悸、心跳加速吗？ |  |  |  |  |  |
| 28.您曾感觉到手脚麻木吗？ |  |  |  |  |  |
| 29.您曾感觉到手脚反应迟钝吗？ |  |  |  |  |  |
| 30.您曾感觉到手脚对冷热不敏感吗？ |  |  |  |  |  |
| 31.您曾有因为治疗引起脱发吗？ |  |  |  |  |  |
| 32.您的手脚出现过红肿、脱皮吗？ |  |  |  |  |  |
| 33.您曾有便秘吗？ |  |  |  |  |  |
| 其他维度 | 一点也不 | 有一点 | 有些 | 相当 | 非常 |
| 34.您的身体状况或治疗过程，造成了您的经济困难吗？ |  |  |  |  |  |
| 总体自我评价维度 | 一点也不 | 有一点 | 有些 | 相对 | 非常 |
| 35.您对现在的治疗状况满意吗？ |  |  |  |  |  |
| 36.您认为自己今天的健康状况好坏程度如何？ | 很差 | 比较差 | 一般 | 比较好 | 很好 |
| 37.您如何评定过去一周中您的整体健康状况？ | 很差 | 比较差 | 一般 | 比较好 | 很好 |
| 38.您现在的健康状况与一年前的健康状况相比变差了吗？ | 很差 | 比较差 | 一般 | 比较好 | 很好 |
